# Social Support and Diabetes Management Among Older American Indians

**DOI:** 10.3389/fpubh.2022.780851

**Published:** 2022-06-21

**Authors:** R. Turner Goins, Molly K. Grant, Kathleen P. Conte, Lisa Lefler

**Affiliations:** ^1^Department of Social Work, Western Carolina University, Cullowhee, NC, United States; ^2^Department of Public Health, DePaul University, Chicago, IL, United States

**Keywords:** older adults, American Indians, social support, type 2 diabetes, in-depth individual interviews

## Abstract

**Objective:**

Greater understanding how relationships that can facilitate or impede type 2 diabetes (T2D) management and control among older American Indian people is an overlooked, yet urgently needed strategy. Thus, we examined social support among older American Indian people in relation to their T2D management.

**Methods:**

During the fall 2015, we conducted qualitative interviews with 28 participants aged ≥ 60 years who were members of a federally-recognized tribe. Drawing upon the buffering and direct effects theoretical models of how social support affects health, we examined transcribed audio recordings of the interviews with a systematic text analysis approach. We used a low-inference qualitative descriptive design to provide a situated understanding of participants' life experiences using their naturalistic expressions.

**Results:**

The mean age of our participants was 73.0 ± 6.4 years with a mean HbA1c of 7.3 ± 1.5. Main social support sources were family, clinicians/formal services, community/culture, and spiritual/God. All four common social support types were represented, namely emotional, instrumental, informational, and appraisal support with most being instrumental in nature. A prominent gender difference was seen with respect to men receiving more instrumental support family/friends support than women.

**Discussion:**

Value orientations among American Indian people often reflect extended social systems and interdependence. A deeper understanding is needed of how social relationships can be better leveraged to aid in effective T2D management among older American Indian people. The development and implementation of evidence-based social network interventions with an assets-based orientation that build upon the cultural value of reciprocity hold promise to improve T2D outcomes of older American Indian people.

## Introduction

### Background

There are three main types of diabetes with type 2 diabetes (T2D) being the most common −90 to 95% people with diabetes have T2D (1). Age-adjusted prevalence of diabetes has experienced a significant increase, rising from 9.5% in 1999–2002 to 12.0% in 2013–2016 in the general U.S. population (2). An examination of the Indian Health Service patient population found a significant increase in diabetes prevalence among American Indian and Alaska Native (AI/AN) people from 2006 to 2013, followed by a significant decrease from 2013 to 2017 (3). Despite this decrease, AI/AN people continue to have the highest rates of T2D in the U.S. Compared to their White counterparts, AI/AN people are twice as likely to have T2D (2) and three times more likely to die from T2D (4). T2D is one of the leading causes of morbidity and mortality among AI/AN people, which increases with age (5), making it a top priority for the Indian Health Service and tribal health care systems. Treating T2D consumes 37% of all medical costs for the Indian Health Service and age-adjusted total health expenditures for persons with T2D is over three times higher than those for persons without T2D (6).

The aging of the U.S. population is one of the main drivers increasing T2D prevalence (7). Among persons of all racial groups and ethnicities aged ≥ 65 years, it is estimated 27% have diabetes while 47% have prediabetes (2). Data from one tribe indicate that 60% of tribal members aged ≥ 65 years had T2D (8). Between 2020 and 2050, the number of AI/AN people who are aged ≥ 55 years is estimated to increase 1.8-fold, 2.2-fold increase among those aged ≥ 65 years and older, and 5.0-fold increase among those aged ≥ 85 years of age (9). Altogether, given the prevalence of diabetes among AI/AN people and the projected increase of older AI/AN people, it is critical the factors that affect older AI/AN peoples' management of diabetes be understood.

There is a growing body of research on the impact of social relationships on the management and control of diabetes. Social support is a broad construct and operationalized numerous ways including interpersonal relationships, the belief and actuality that one is cared for, and social resources received as part of these relationships (10). Social support measurements can include the structure, processes, and functions of relationships. There is mounting evidence that social support may figure importantly in T2D management. Recent findings suggest that greater positive social support promotes T2D management (11, 12) whereas negative social support can have the opposite effect (13–15).

Social support falls within a larger conceptualization of the notion of connectedness in Indigenous populations. Indigenous cultures have strong values around social connectedness and reciprocity (16). Recently, an Indigenous oriented framework has been developed identifying the mechanisms of connectedness and reciprocity that exists specifically between child and collective wellbeing. The mechanisms were identified through the existing literature pertaining to Indigenous populations worldwide and included family, community, environment, intergenerationality, and spirituality (17). Although research is growing on this topic, more remains to be understood in this regard among AI/AN people despite connections with others being a core indigenous cultural value (18, 19).

Findings from a few studies with AI/AN people suggest that improving our understanding of social support in the context of T2D management may lead to important clinical and public health benefits. A study of diet and nutrition among Navajo people revealed improved outcomes—notably lower HbA1c levels—among tribal members with T2D who jointly prepared meals with family members (20). A study among Alaska Native people with T2D found that social support was an important element in managing their T2D. Specifically, participants cited social support as a key source of encouragement for dietary adherence and coping (21). Third, the evaluation of the Special Diabetes Program for Indians Diabetes Prevention demonstration project—a large, nationwide translational initiative involving 3,135 AI/AN people with prediabetes—found a significant association between negative family support and less weight loss, and between positive family support and greater weight loss at both baseline measurements and over the course of intervention (22). More recently, a study with American Indian adults from five tribal communities in the Upper Midwest found that general and T2D-specific support was associated with increased T2D self-efficacy (23).

While this prior research is important, it is surprising there has not been closer attention to social support as an existing strength of AI/AN communities, making it likely a promising focus for programs and/or interventions designed. Prior analyses of the study data explored the primary research questions (24–26) among a sample of older American Indian people. While conducting these earlier analyses, it was observed that social relationships and support were frequently discussed by participants regarding T2D management which led to an examination of social support in more detail. Thus, for this study, the qualitative interview transcripts were reexamined for the role and nature of social support in the context of daily T2D management—specifically, the sources and types of social support received and how these may differ by gender.

### Conceptual Framework

Research regarding social support is vast with respect to health outcomes. In this context, social support refers to as any process through which social relationships facilitate health and well-being (27). A well-cited definition of social support categorized the functional content of relationships in four types: Emotional, instrumental, informational, and appraisal (28, 29). Emotional support refers to things people do that make us feel loved and cared for, that bolster our sense of self-worth. Such support can include talking over a problem or providing encouragement. By contrast, instrumental support refers to the various types of tangible aid provided by others, including yard work and transportation. Third, informational support is help others may offer by providing advice, suggestions, or information. Lastly, appraisal support involves providing affirmation, feedback, social comparison, and information for self-evaluation (10).

Researchers have examined the impact of social relationships on health for over 100 years notably dating back to Durkheim's work (30), who found suicide to be more common among persons with fewer social connections. Since then, several theoretical models have been developed in an attempt to disentangle the complex association between social relationships and health (31–33). Broadly, there are two theoretical models that delineate how social support influences health. The first is a buffering model that suggests social support protects against the negative effects of stressors. The second is a direct effects model that contends social support can be the beneficial absence of stressors. Depending on the circumstances, social networks can both negatively and positively impact health and health-related behaviors. In the present study, these models are not tested but rather drawn upon to inform an interpretation of what study participants shared with respect to their experiences with social networks specific to their T2D management. Social support can affect engagement in T2D management by influencing emotions, cognitions, and behaviors (34). Social relationships can also directly influence such health-related behaviors such as diet, exercise, smoking, sleep, and adherence to medical regimens (35).

## Methods

### Research Design

This study was a secondary analysis of qualitative data collected for a larger study of older tribal members that examined their perceptions and experiences in managing T2D. The research questions and design of the primary study emerged from initial discussions with tribal leaders and, later, with the study's Community Advisory Board (CAB). The tribe's institutional review board (IRB), tribe's health board, and tribal council approved this project.

### Participants

Potential participants were identified from the surviving *[study name blinded]* participants, a prior study conducted with the same tribe. The first author [RG] was the Principal Investigator of the [Native Elder Care Study] which included 505 participants who were interviewed between July 2006 and August 2008. Study eligibility included tribal enrollment, an age ≥ 55 years, noninstitutionalized, cognitively intact, and residing in the tribe's service area. More detail about the [Native Elder Care Study] methodology is described elsewhere (36).

In the present study, eligibility included being an original participant in the *[Native Elder Care Study]*, residing in the tribe's service area, and having self-reported T2D. Drawing upon existing records of *[study name blinded]* participants, letters were sent to those who had indicated they had T2D and who were still alive. The participant information was linked with public state death records to remove deceased participants from the mailing list. After two mailings, 47 interested participants responded by calling the Principal Investigator who confirmed their study eligibility; 28 ultimately participated.

### Data Collection

In-person data were collected with semi-structured, in-depth qualitative interviews using an interview guide. The guide was initially developed and piloted with a convenience sample of six tribal members (four women, two men) who were aged ≥ 60 years and had self-reported T2D. The project's Principal Investigator conducted these piloted interviews accompanied by the project's graduate research assistant for training purposes. The interview guide was revised based on these pilot interviews and input provided by the study's CAB.

The final interview guide consisted of 19 main questions with probes (See Table 1). Each participant's age and gender were determined through the *[Native Elder Care Study]* data; current marital status was ascertained during the interview. Participants' most recent HbA1C laboratory results were obtained from the tribe's hospital records. The HbA1C test reflects the average of a person's blood glucose levels over the 2–3 months prior to interview and is represented as a percent. While HbA1c values vary by demographic characteristics, generally < 5.7% was considered normal, 5.7–6.4% considered prediabetes, and ≥ 6.5% considered T2D (37).

**Table 1:** Interview questions.

1. How long have you had type 2 diabetes?
2. What do you think about having diabetes?
3. What is different between those who get diabetes and those who don't in the [tribe name removed]?
4. What do you think caused your diabetes?
5. How confident do you feel that you can successfully manage your diabetes on a scale from 1 to 10 with 1 representing no confidence and 10 representing the greatest confidence?
6. How do you manage your diabetes on a daily basis?
7. Overall, would you say that your diabetes is well-managed or poorly managed?
8. Can you share a time when you knew your diabetes was not as well managed as it could be?
9. What are the primary factors or reasons why you say your diabetes is well-managed/poorly managed?
10. Among those who have diabetes in the tribe, what do those people with well-managed/poorly managed diabetes do differently than you?
11. Who helps you with your diabetes?
12. What is the value in managing your diabetes well?
13. What are the consequences of not managing your diabetes well?
14. How do your feelings, such as feeling down, or tired, or out of energy, affect your ability to manage your diabetes?
15. What is it that you wish others understood about your diabetes?
16. Is there anything you need that you don't already have to better manage your diabetes?
17. Other than what the doctor has told you and what you have learned from experience, is there a [tribe name removed] way to deal with your diabetes?
18. In general, what are the factors that you believe that contribute to good diabetes control?
19. Are there any other thoughts you have about your diabetes and your ability manage it that you would like to share?

During fall 2015, 20 interviews were conducted by the project's Principal Investigator and eight interviews were conducted by a graduate research assistant trained for this study. Each interview was conducted in-person at a private location of the participants' choice. Most interviews were conducted in the participants' home; one interview was conducted in the Principal Investigator's office; the remaining interviews were conducted in a room at a tribal building. Interviews lasted an average of 48 min, were digitally audio-recorded, and professionally transcribed. The transcriptions were audited by three of the study's investigators for completeness and accuracy. The study acquired written informed consent and permission to obtain hospital laboratory HbA1c results from the participants who received $75 for their contribution.

### Analyses

The secondary analysis presented here was performed using thematic analysis which is a qualitative descriptive approach that relies on a low-level interpretation to provide a situated understanding of participants' life experiences using their naturalistic expressions (38, 39). The low-level interpretation relied on verbatim accounts of what participants offered and minimized the extent to which the researchers reconstructed what participants shared. Individual transcripts and team debriefing recordings formed the data for the analyses. Thematic analysis was used, which is an interpretive process involving systematically searching the data to identify patterns to generate an illuminating description of the phenomenon (40). The research team engaged in a mixed inductive and deductive thematic analysis (41) through processes led by the Principal Investigator who is a senior researcher [*RG*]. Although the interview guide only included one question about the involvement of others in the participants' daily T2D management, participants discussed social support in a variety of ways throughout the interview. Thus, team members used the full transcripts for this analysis.

The team consisted of four investigators with social work *[MG]*, public health *[KC]*, gerontology *[RG]*, and anthropology *[LL]* backgrounds. Having a multidisciplinary analytic team helped contribute to investigator triangulation (42) in determining where and how social support was involved with the participants' T2D management and thus strengthened credibility of the analyses (43). The team-based analytic process consisted of reading fully each transcript and recording detailed notes and examples of how social support was discussed by each participant. Transcripts were analyzed first to understand everyday participant experiences as a whole (44) and then into individual stimuli, behaviors, or both with a line-by-line approach to *in vivo* coding using participant language with respect to relationships and their T2D. After coming together to discuss the initial analysis, the team identified that social support was commonly discussed in terms of *who* provided support to participants and *in what ways*. Therefore, it was decided to conduct a directed content analysis (45) to identify sources of support among the participant sample and types of support.

Three of the authors *[RG, MG, KC]* then reread each transcript to identify sources and types of social support. Sources of support were identified then organized into categories as reflected in the data, following the four types of functional content of relationships as established in the literature (28, 29). The data were sorted by gender for both type and source of support to explore and identify patterns and the intersection of types and sources were determined. The data were also ordered to examine trends by other qualities, such as whether participants reported receiving social support or not and whether participants had someone to exercise with or not, but no meaningful differences were detected. Regular meetings were held over a 9-month period to review and interrogate findings, including investigator triangulation (46) and use of an iterative design with emergent findings (47). An audit trail of team discussions, theme development, and the refinement of the analytic framework was maintained through audio recordings and note-taking.

## Results

There were 28 study participants with a mean age of 73.0 ± 6.4 years. Slightly more than half (57.1%) of the participants were female and 50.0% were married. The mean HbA1c was 7.3 ± 1.5.

### Sources of Support

Participants discussed four themes as sources of support, including family/friends, clinicians/formal services, community/culture, and spiritual/God. The theme family/friends included others who the participant was related to or a friend of the participant. Clinicians/formal services included tribally based providers such as physicians, nurses, nutritionists, and certified diabetes educators. This theme also included non-clinical tribal programs such as the Senior Center. The theme community/culture was a feeling of community belonging and/or specific community-based cultural practices, functions, and activities. The fourth theme, spiritual/God included spiritual-related practices and beliefs, a relationship with a higher power entity, and the participants' congregation or group of individuals in which they worship with. Lastly, a few participants spoke about the lack or absence of support. Exemplar quotes are presented in Table 2.

**Table 2:** Exemplar quotes by support source.

**Females**	**Males**
**Family/Friends**	
I need to extend my walking some, but we would go down this, way, and down that way, and over through the field, and back around. We try to do that every evening. My husband and I. [F07].	We just started eating less. She obviously cut back on the–when she bakes a cake she cuts back–she don't put as much sweetening in it. She makes more of a diabetic cake when she does it because she herself don't like too much sweetness. [M09].
My daughter takes care of the shots and my pills. She does all of that for me. [F18].	As I say, my oldest daughter, she was a nurse and she tells us things to do. She writes down things that I should do as a diabetic. My wife goes along with that and tells me, “Your own daughter knows that you've got the diabetes and she's trying to help you.” [M12].
**Clinicians/Formal services**	
This (tribe senior center) is my day-to-day social interaction with people my own age and it's always positive. I grew up here as a child so most of these people I've known all my life and they keep me informed of what's going on in the community because where I live…we visit and talk and we're just glad to see each other every day. [F26].	Yeah—it's very cost-effective…If you can just go to the (tribal) hospital up there and say; look, I need some lancets and I need some strips and blah, blah, blah. [M32].
We have a nurse who comes around fills our pill box [F24].	Well, the tribe diabetes clinic, they kind of help me manage and control my diabetes [M27].
**Community/Culture**	
Conditioning. I come from an old traditional family. In fact, we were discussing that yesterday. My Indian grandparents didn't speak English. He was a tribal medicine man. So I grew up with a lot of herbs, the teas, the practices and just growing up you keep them, you maintain them, which is what the four older ones of us did because we were raised mainly in that atmosphere where you didn't fry potatoes [F30].	The tribe come up here about a month ago with – they was about 15 to 20 people. It was time for the mower to come and mow, but they beat the mower to it. There was about five of them had weed eaters and they started way down there at the garden, come up here weed eating…and done all the weed eating. I had some rocks laying back here behind the house. We told them that. They got it and they made flower beds… They fixed the front of the house right along through here. They just done everything and we appreciated it [M12].
And they are always harping about eating the right things, and then they'll have all these Indian dinners served for benefits, and there's always the wrong type of food to eat [F07].	I mean, if you're a member of a society like our society, like Cherokee, you're not as much as maybe a suburban city or some people in the outside world are not as concerned as what we are about thinking about not just ourselves but our family, our community, the whole society [M29].
**Spiritual/God**	
I can get down, but then always God reminds me in a gentle voice, sometimes a little bit stern, now you just stop it. Just stop it. You've got to keep moving. Keep moving [F18].	I was bad before I got saved and got into church [M14].
So, it's (church) another community, but it's a familiar community and so all of that really takes up most of my days, most of time [F26].	I think that God gives the doctors knowledge to keep us going, and if we don't take that treatment the doctor prescribed for us, it's like saying, “God, you don't know what you're talking about.” [M37].
**Absence/Lack of**	
I have to do it (exercise) by myself [F22].	Maybe more encouragement from my healthcare providers and from family members–they don't always encourage each other like we should. Sometimes you don't know how to encourage somebody. [M09]
They can get it at the dialysis center, but they don't have any support when they get home because when they get home it's fixed the way that they're not supposed to have it [F30].	No–not on diabetes. I don't know if we've really took it seriously enough to dedicate a certain part of the time for a men's group to get together and discuss and help support each other [M09].

Overall, most of the support came from family/friends followed by clinicians/formal services. Discussions of family/friends were usually about interactions with their spouse and adult children. Regarding clinicians/formal services, participants reflected on the kindness and/or helpful advice received from tribal clinicians as well as tribal programs. Participation in tribal programs such as the senior center, fraternal organization, or a support group provided an opportunity to connect with others. Support from community/culture presented itself in the form of identifying as a member of the tribal community, attending community-wide events, the value of caring for each other and reciprocity, and shared teachings of traditional medicine. Participants also noted the importance of community-level activities that reinforced connectedness; yet, such events were also known for providing food choices not conducive for T2D management. The theme of spiritual/God primarily involved the participant's relationship with a higher power most often referred to as “God.” Also, this fourth theme included fellow worshipers or congregation members. Most of the participants had at least one source of support although some participants reported an absence or lack of support with respect to T2D management. When asked, a few participants indicated that they did not have anyone to socialize, eat, or exercise with and/or did not have someone to provide them relevant tangible aid.

Regarding gender differences, men tended to discuss the support of family/friends more than women. The family/friends support that those men spoke more of was predominantly provided by a woman either their wife or an adult daughter. There were no observable gender differences in other kind of support sources.

### Types of Support

Evidence of the four types of support was examined among study participants including emotional, instrumental, informational, and appraisal support (28, 29). Exemplar quotes are in Table 3. All four types of support were present among the study participants. Overall, there was limited discussion of emotional support among participants, but the emotional support received was in the forms of feelings of trust and caring. Further, the quote provided from participant F34 was classified as emotional although it mentions the provision of food, which could be considered instrumental support. Providing food in the participating community is more than simply obtaining nourishment but it also takes on a strong cultural way of showing emotional support. Thus, it is unclear if the lack of emotional support discussed was attributable to participants not having this type of support, were less likely to discuss it due to the due to lack of specific questions on it, or do not usually frame social support as emotional *per se*.

**Table 3:** Exemplar quotes by types of social support.

**Females**	**Males**
**Emotional: Expressions of love, empathy, trust, caring, and encouragement**	
I have a sister that has it, and we visit, and talk about it [F08].	She gets upset when I don't exercise [M37].
My daughter-in-law will help; she's always cooking something and wanting me to eat with her [F34].	I belong to the Legion and we have a little meeting on Thursday mornings. A bunch of us if we go there sometimes and we eat. It's a monthly meeting for all the Legionnaires but on Thursday it used to be a gab session [M25].
**Instrumental: Tangible aid and service**	
Yeah, I like her (physician). She takes good care of me [F26].	Well, like I said–there again, I go back to my wife. She was a diabetic teacher. And she sort of fixes meals that line up with that [M14].
My husband got me things (Indian medicine) the first time and it kept it down, but like I said, I hate to deplete the plants, because you have to take it all the time, too [F10].	My wife doles out my pills, so I take those religiously. I haven't missed a single one. She, as I mentioned, checks my blood sugar level [M29].
**Informational: Advice, suggestions, and information**	
That's when I smoked and the doctor told me, not a cigarette–he said, “How much you smoke?” And I said, one a day. He said, “No more.” Okay. I never picked up another cigarette [F11].	My step daughter's beginning to notice that. She said, “Dad, better wake up. Better move around. Quit sitting in one place” [M23].
I had gone to the hospital, I had an appointment with the nutritionist and we talked and everything...we had a really long discussion, about diabetes, you know, and the effects, and everything about it. And, nobody had ever told me anything like that before…Oh, yeah, it was really helpful [F34].	As I said, being familiar with that, I still didn't go until finally my wife made me go to one of my specialists [M29].
**Appraisal: Affirmation, feedback, social comparison, and self-evaluation**	
My mom had it, and so she was real bad. So I was scared to death to have it. But it hasn't been–if you watch what you eat [F08].	There's tingling and burning in your feet. That still concerns me but I got my confidence somewhat from my mother. She'd been on 1–2 shots a day for 15 years. She changed her living habits and her eating habits and she came completely off of them after 15 years. So I know that I can do it if I've just got the will power. I was inspired by her...I guess that was what started the hope that if you have the dream don't let nobody take it away from you [M09].
Well, my brother, like I said, his really gets him down. But they told him that he was going to have to go on dialysis and this was about maybe 8 years ago. And he's like, he's not gonna take this route. So he's real strict on his diet and does the carb count and this and that and whatever. And he's managed to stay off the dialysis and he's still alive so I can see the benefits of being really strict with yourself and watching your diet. I can see a positive there, as far as he's concerned [F31].	I had in church, prayer meetings we called it, with people...and we've run into differents of places that people didn't have legs. They smoke. They do everything. They don't eat right. They don't take care of themselves. I mean, I see this, you know, myself. But when we went into these houses, I seen this one lady, she was in the hospital bed, with–we didn't even know that she was in this shape when they asked us to come to their house and have prayer meeting. Her legs, both legs, was amputated...You can see for yourself that it's not good [M12].

Instrumental and informational types of support were the most discussed among the four types. Instrumental support included specific T2D management advice and information, medication management, food shopping and preparation, and exercise encouragement and/or accompaniment. Much of the informational support was specific T2D management information provided mostly from the clinicians accessed through formal services or a family member with relevant clinical training. Also, informational support from family members who encouraged the participant to engage in T2D management behaviors such as exercise or to obtain medical care. Appraisal support was discussed in the context of participants comparing themselves to family or community members who had or also have T2D.

With respect to gender differences, although there was relatively limited discussion of emotional support, it was disproportionately referenced by women participants. Women mostly received their emotional support from other women such as friends or adult children. Men more often discussed receiving instrumental kinds of support than women–mostly for dietary-related needs such as food shopping and meal preparation. There were a few men in which their T2D management was entirely taken care of by their spouse, including food shopping, meal preparation, scheduling routine medical appointments, and medication management. There was one woman whose medication management was handled by her spouse. No notable gender differences were observed regarding receipt of informational or appraisal support.

Table 4 illustrates how sources of support and types of support intersect in the study data. Family/friends provided all four types of support. Clinicians/formal services provided emotional, instrumental, and informational support. Community/Culture provided instrumental, informational, and appraisal support and Spiritual/God provided emotional and informational support. Emotional and instrumental support were provided from three sources, informational was provided from all four sources, and appraisal was provided by two sources.

**Table 4:** Intersection of social support sources and types.

	**Emotional**	**Instrumental**	**Informational**	**Appraisal**
Family/Friends	✓	✓	✓	✓
Clinicians/Formal Services	✓	✓	✓	
Community/Culture		✓	✓	✓
Spiritual/God	✓		✓	

## Discussion

This study adds to the limited contemporary literature on the relationship between social support and T2D among American Indian peoples. This is the first study to examine sources and types of social support in the context of T2D management among older American Indian people. Four themes regarding support sources were identified: Family/friends, clinicians/formal services, community/culture, and spiritual/God. Additionally, all of the four common types of support established in the literature were present with most support being instrumental in nature. Although there has not been other research with the goal of identifying sources of support among older American Indian people with T2D, prior work has identified the importance of family with respect to T2D management (20, 22). Collectively, earlier research shows how positive family support helps with T2D management while negative family support hurts these efforts. A qualitative study with Alaska Natives that did not exclusively focus on family as a source of support examined factors that have a positive influence on T2D management. Social support from other people with T2D was a factor participants reported as something that facilitated their T2D management (21).

The most prominent gender difference found was men receiving instrumental support from family/friends more than women. Similarly, a recent literature review found that overall, women receive little or no spousal support with their T2D (48). Further, research with American Indian people who had T2D found that being female was negatively associated with receiving T2D support (23). Yet, interestingly, quantitative findings with the larger *[Native Elder Care Study]* sample showed no significant gender differences in the *types* of social support received, including instrumental support as measured by the Medical Outcomes Study–Social Support Survey (49).

These findings indicate that there are gendered differences in how participants think about, value, and describe social support. Prior research has established that gender differences in caregiving participation and care patterns are often attributed to conventional gender role norms. Such conventional roles allocate household management, childrearing, and care of sick family members to women while men are socialized to pursue a livelihood in the working world (50). Caring for a husband with a chronic disease in need of daily management, therefore, appears to be an extension of women's social roles (51). Researchers that examined spousal support and dietary adherence for T2D management found that these conventional gender roles appear to benefit the man and not the woman (48). Women study participants tended to talk about emotional types of support compared to men. This finding aligns with researchers who maintain that women value close relationships for their emotional features while men mainly conceptualize close relationships in terms of their instrumental features (52). As this may relate to T2D management and control, other research with older adults found that women and not men had a significant positive relationship between receipt of emotional support and engagement in physical activity (53).

Given these data and findings, there are active mechanisms in which relationship characteristics facilitate or impede T2D management and control that present opportunities for programs and/or interventions. For instance, family/friends as well as community/culture both played roles in available choices and dietary habits. Family/friends and community-wide events influenced food choices and preparation. In some cases, the influence was positive and facilitated T2D management while in other cases, the influence was negative and impeded T2D management. Here, positive T2D supportive efforts would involve the larger family unit that surrounds someone with T2D in providing information as to how food affects management and control. Also, given the value of community-wide events in reinforcing connectedness, targeting the planners of such events regarding the importance of providing healthier food options, would likely be beneficial.

The influence of relationships on health outcomes can be complicated, and the social and cultural contexts need to be considered. American Indian families often reflect lateral relational behavior rather than autonomy and independence with extended social systems. Family is defined by its relation, connection, and alignment to life (54). Since this study's participants resided in their tribal community, the notion of family commonly extends beyond the conventional nuclear family. Yet, it would be helpful to understand the extent to which sociocentric vs. egocentric orientations exist and how they relate to T2D management. In the early 1900s, in the study's participating tribe, young adults would often continue to live with or near parents' or other older relatives' households and share resources. Extended families, or two or more nuclear families, made up of multiple generations sharing living environments. Grandmothers were at the core of these kin networks and, to a lesser extent, families in this tribe continue to live in extended family groupings (55). However, it is important not to make assumptions about how social systems are functioning in a tribal community as to appreciate that notions of individuality also exist. Thus, to develop programs and interventions that leverage social support for better T2D related outcomes, a deeper understanding would help researchers appreciate how relationships are affecting health and the extent to which social systems vary among American Indian people and communities.

It is reasonable to expect that social systems have important implications for preventing and treating chronic diseases such as T2D in tribal communities. A qualitative study with older American Indian peoples found that participants believed that having a support system was key to preventing or managing T2D (56). Given the value of social connections among AI/AN people, promising future efforts would seek to understand how social support can be leveraged regarding the prevention or treatment of T2D. Research with other racial/ethnic populations have found improvements in self-management and biomedical outcomes as a result of formal interventions primarily with strangers (57) while it is hypothesized that naturally occurring relationships hold far greater potential to influence health outcomes.

Increased understanding of how relationships can aid in effective T2D management can inform more appropriate approaches to intervention which can support others working to adopt new health behaviors and/or aid in health behavior maintenance. Research has shown that among persons with poorly controlled T2D, having both a caregiver and social support for T2D can be helpful for medication adherence (58). For at least several decades, the research literature has established robust and consistent associations between various components of one's social network and health (59–62). Armed with such knowledge, more informed decisions can be made as to where to focus intervention efforts. Also, the findings seem to suggest that interventional efforts need to consider that many women are likely taking on a lot of the day-to-day instrumental support tasks for the husband with T2D. These women may or may not have T2D themselves and would likely benefit from an assessment of their social support needs.

There are several study limitations that warrant acknowledgment. This study was conducted with one tribal community and given the diversity across American Indian communities with respect to culture, language, and beliefs, future research is needed with other American Indian tribes in similar and other geographic regions. Second, these data were collected through a single round of interviews. Thus, it is likely that if more than one interview per participant were conducted, greater rapport would have been established, potentially yielding more information regarding the role of social support with respect to their daily management of their T2D. Although member checking was not conducted with the study participants, the transferability of these findings was enhanced by the context validation received through the input of the larger project's CAB. Lastly, the study is limited in that it was a secondary analysis of a study not intentionally designed to investigate social support and so the data collected on this topic may be incomplete despite it featuring heavily in the participant narratives. It may be, for instance, that males primarily discussed instrumental types of support because the only question focused on this type of support.

The term social network refers to the web of relationships surrounding individuals with receipt of social support being one of the valuable functions of relationships (10). Prior research has been unable to identify social network characteristics that enhance T2D management and control (63, 64). The inability to identify the social network characteristics can be attributed to inadequate research attention to deeper possibilities. Despite the limitations, this current study provides some insights into the role of social support with respect to T2D management among older American Indian people. With an assets-based orientation, there are needs to develop and implement evidence-based interventions that build upon the cultural value of social connectedness and reciprocity designed to improve T2D outcomes of older American Indian people.

## Data Availability Statement

The raw data supporting the conclusions of this article will be made available by the authors, without undue reservation.

## Ethics Statement

The studies involving human participants were reviewed and approved by Western Carolina University's Institutional Review Board and by the participating tribe's medical institutional review board.

## Author Contributions

RG and MG contributed to conception and design of the study. RG, MG, LL, and KC contributed to the analyses and writing the manuscript. All authors read and approved the submitted version.

## Funding

The study was funded by the National Institutes of Health and the Indian Health Service through the Native American Research Centers for Health [U261IHS0078].

## Conflict of Interest

The authors declare that the research was conducted in the absence of any commercial or financial relationships that could be construed as a potential conflict of interest.

## Publisher's Note

All claims expressed in this article are solely those of the authors and do not necessarily represent those of their affiliated organizations, or those of the publisher, the editors and the reviewers. Any product that may be evaluated in this article, or claim that may be made by its manufacturer, is not guaranteed or endorsed by the publisher.
